# Transformer-based multimodal precision intervention model for enhancing diaphragm function in elderly patients

**DOI:** 10.3389/fncom.2025.1615576

**Published:** 2025-08-18

**Authors:** Ma Xinli, Zhao Jie, Yan Ming, Zhang Yanping, Li Fan, Jia Jing, Ding Lu

**Affiliations:** ^1^Critical Medicine Department, The Second Hospital of Jilin University, Changchun, Jilin, China; ^2^Nursing Department, The Second Hospital of Jilin University, Changchun, Jilin, China; ^3^School of Nursing, Jilin University, Changchun, Jilin, China; ^4^General Surgery Department, The Second Hospital of Jilin University, Changchun, Jilin, China

**Keywords:** transformer models, multimodal data integration, diaphragm dysfunction, mechanical ventilation, predictive modeling, critical care applications

## Abstract

Diaphragm dysfunction represents a significant complication in elderly patients undergoing mechanical ventilation, often resulting in extended intensive care stays, unsuccessful weaning attempts, and increased healthcare expenditures. To address the deficiency of precise, real-time decision support in this context, a novel artificial intelligence framework is proposed, integrating imaging, physiological signals, and ventilator parameters. Initially, a hierarchical Transformer encoder is employed to extract modality-specific embeddings, followed by an attention-guided cross-modal fusion module and a temporal network for dynamic trend prediction. The framework was assessed using three public datasets, which are, the MIMIC-IV, eICU, and Chest X-ray. The proposed model achieved the highest accuracy (92.3% on MIMIC-IV, 91.8% on eICU, 92.0% on Chest X-ray) and surpassed all baselines in precision, recall, F1-score, and Matthews correlation coefficient. Additionally, the model's probability estimates were well-calibrated, and its SHAP-based explainability analysis identified ventilator volume and key imaging features as primary predictors. The clinical implications of this study are significant. By providing precise and interpretable predictions, the proposed model has the potential to transform critical care practices by offering a pathway to more effective and personalized interventions for high-risk patients.

## 1 Introduction

Diaphragm dysfunction constitutes a significant clinical challenge in the management of elderly patients, particularly those who require mechanical ventilation (Kaya and Iscimen, 2025). As the primary muscle responsible for respiration, the diaphragm's optimal function is crucial for effective breathing and gas exchange (Lessa et al., 2016). However, in elderly populations, age-related muscle degeneration, comorbidities, and the detrimental effects of prolonged mechanical ventilation contribute to a high incidence of diaphragm dysfunction (Powers et al., 2009; Siniscalchi et al., 2024). This dysfunction not only compromises respiratory efficiency but also elevates the risk of extended intensive care unit (ICU) stays, ventilator-associated complications, and overall mortality (Ganatra and Varisco, 2019).

Mechanical ventilation, while lifesaving, presents several inherent challenges that complicate the assessment of diaphragm function (Ratano et al., 2024). Ventilator-induced diaphragm dysfunction (VIDD) is a well-documented phenomenon, wherein the act of mechanical support leads to diaphragmatic atrophy and impaired contractility (Vassilakopoulos and Petrof, 2004). Traditional methods of diaphragm evaluation, such as ultrasound imaging and electromyography, are often operator-dependent and limited in their capacity to capture the complex, dynamic interplay between patient physiology and ventilatory support (Laghi et al., 2021; Sarwar et al., 2024). These limitations hinder timely intervention and may result in suboptimal management strategies in critically ill patients.

Current clinical practices and predictive models for assessing diaphragm function exhibit significant limitations (Llamas-Álvarez et al., 2017). Most conventional models rely heavily on static snapshots of clinical parameters, neglecting the temporal evolution of respiratory mechanics (Hsia et al., 2023; Hantos, 2021). Additionally, many existing methods fail to integrate the heterogeneous nature of available clinical data, ranging from imaging and physiological parameters to ventilator settings, thereby restricting their predictive accuracy (Howroyd et al., 2024). The reliance on isolated, single-modality data often results in models that are unable to capture the nuanced and dynamic patterns inherent in patient responses during mechanical ventilation (Adibi et al., 2025). Consequently, clinicians are left with decision-support tools that provide limited insights and lack the robustness necessary for effective intervention planning.

The primary research question of this study is: Can a Transformer-based multimodal model that simultaneously processes imaging, physiological signals, and ventilator metrics accurately predict diaphragm dysfunction in elderly patients undergoing mechanical ventilation and facilitate personalized intervention planning? Surpassing current methodologies, which typically concentrate on individual data streams or static snapshots, the proposed framework distinctively integrates hierarchical Transformer encoding, cross-modal attention fusion, and temporal modeling. Additionally, it offers interpretable explanations to provide real-time, data-driven decision support.

Despite advancements in machine learning for critical care (Zhang et al., 2024; Biesheuvel et al., 2024), a significant research gap remains: no current methodology concurrently addresses the temporal dynamics of diaphragm function, integrates multimodal clinical data at scale, and ensures interpretability for clinicians. Previous studies have either concentrated on individual data streams (e.g., imaging alone) or utilized traditional time-series models that do not capitalize on recent deep learning architectures. Notably, Transformer-based methods, which are proficient in modeling long-range dependencies in heterogeneous data, are underutilized in this field (Kim et al., 2025). This gap highlights the necessity for a comprehensive, real-time predictive framework capable of integrating imaging, physiological signals, and ventilator metrics to inform personalized interventions.

The emergence of artificial intelligence (AI) and deep learning presents a transformative potential to address these challenges. In recent years, deep learning architectures, particularly those utilizing Transformer models, have demonstrated significant success in processing complex and multimodal data (Xu et al., 2023; Zhang et al., 2023; Mostafaei et al., 2024). Transformer models, characterized by their multi-head self-attention mechanisms, excel at capturing both local and global dependencies within data sequences, rendering them highly suitable for time-series analysis in critical care environments (Ma et al., 2019b; Yamazaki, 2024). The capability of these models to integrate information from diverse sources, such as imaging data, clinical text, and physiological signals, offers a unique opportunity to develop more comprehensive and accurate predictive models for diaphragm dysfunction.

This study presents a novel Transformer-based multimodal framework designed to integrate diverse clinical data, thereby addressing the previously identified limitations. The framework employs a hierarchical Transformer encoder for feature extraction, which is succeeded by an attention-guided fusion module that aligns imaging and textual features. Subsequently, a temporal network captures dynamic patient trends, facilitating predictions that support early and personalized interventions.

The contributions of this work are 3fold.

- First, an AI-driven framework is developed to predict diaphragm dysfunction in elderly patients under mechanical ventilation by integrating diverse data sources.
- Second, the clinical utility of the framework is validated against state-of-the-art methods using rigorous performance and responsiveness metrics.
- Third, the framework's capacity for personalized intervention planning is demonstrated, showing how dynamic ventilator adjustments mitigate diaphragm atrophy and improve weaning outcomes.

The rest of this work is organized as follows. In Section 2, a comprehensive review of the literature on diaphragm dysfunction and mechanical ventilation and transformer models is presented. Section 3 describes the data processing strategy used in this work. The methdological description of proposed work is presented in Section 4. In Section 5, the model training and validation criteria are described. The evaluation porotocols are presented in Section 6. The comprehensive presentation of the experimental results is given in Section 7. Finally, the conclusions are stated in Section 8.

## 2 Literature review

### 2.1 Diaphragm dysfunction and mechanical ventilation

Diaphragm dysfunction in patients undergoing mechanical ventilation arises from a multitude of interconnected mechanisms. Prolonged mechanical ventilation can result in VIDD, which is characterized by diaphragmatic atrophy and impaired contractility (Panelli et al., 2024). This condition is attributed to reduced diaphragmatic activity, oxidative stress, and the activation of proteolytic pathways, ultimately leading to muscle fiber degradation and a decreased capacity for force generation. Scott K Power (Powers, 2024) emphasized that controlled mechanical ventilation can rapidly induce diaphragmatic atrophy and weakness due to inactivity and oxidative stress. Furthermore, sepsis and systemic inflammation exacerbate diaphragmatic weakness by promoting protein degradation and mitochondrial dysfunction.

Diaphragm dysfunction exerts a significant impact on patient outcomes, particularly in relation to weaning from mechanical ventilation (Battaglini and Rocco, 2024). Patients with diaphragmatic weakness frequently experience prolonged weaning periods, increased rates of extubation failure, and elevated mortality (Yayan and Schiffner, 2024). Research has demonstrated that diaphragm weakness is associated with extended durations of mechanical ventilation and prolonged ICU stays, thereby increasing healthcare costs and resource utilization. For example, a study by Jung et al. (2016) found that diaphragm dysfunction in ICU patients was linked to higher rates of extubation failure and prolonged mechanical ventilation.

### 2.2 AI in critical care

AI has been increasingly integrated into critical care to enhance patient monitoring, predict clinical deterioration, and support decision-making (Yoon et al., 2022; Bhakar et al., 2023). AI applications encompass predictive analytics for sepsis detection, mortality risk assessment, and optimization of treatment protocols (Yang et al., 2023). Machine learning models have been employed to analyze complex datasets, facilitating early detection of patient deterioration and informing therapeutic interventions.

In the realm of respiratory care, AI models have been developed to predict respiratory failure, optimize ventilator settings, and assess readiness for weaning (Ahmed et al., 2025). These models utilize patient data, including vital signs, laboratory results, and ventilator parameters, to forecast respiratory decline and guide clinical decisions (Liao et al., 2021). For example, AI algorithms have been applied to predict extubation outcomes by analyzing patterns in respiratory parameters and patient characteristics (Torrini et al., 2021).

Recent investigations have underscored the efficacy of hybrid and metaheuristic-tuned AI models in the realm of medical diagnostics. A notable example is a hybrid convolutional neural network (CNN) integrated with XGBoost, whose hyperparameters were optimized using a modified Arithmetic Optimization Algorithm, achieving an impressive accuracy of 99.39% on a balanced COVID-19 chest X-ray dataset comprising 12,000 images (Zivkovic et al., 2022). In the domain of audio-based respiratory condition classification, the use of CNNs, XGBoost, and AdaBoost, fine-tuned by a modified Particle Swarm Optimization, yielded an accuracy of 98.14% in binary detection and 81.25% in multiclass scenarios (Purkovic et al., 2024). Furthermore, a metaheuristic-optimized Long Short-Term Memory network applied to gait time series data demonstrated an accuracy of 89.92% in detecting Parkinson's disease (Markovic et al., 2025). Similarly, a CNN designed for respiratory condition detection from audio, optimized by a bespoke metaheuristic algorithm, achieved 93% accuracy in binary classification and 75% in multiclass identification (Bacanin et al., 2024a). Additionally, anomaly detection in ECG signals using Recurrent Neural Networks, optimized with the Crayfish Optimization Algorithm, demonstrated superior performance in the early detection of cardiovascular disorders (Jovanovic et al., 2023).

Another recent research has investigated the integration of metaheuristic optimization with deep learning to enhance diagnostic accuracy in complex biomedical applications. For example, Bacanin et al. (2024b) developed a model for detecting Parkinson's disease by combining a deep Long Short-Term Memory (LSTM) network with a modified metaheuristic algorithm for hyperparameter tuning. This study utilizes gait time-series data, employing the metaheuristic to optimize LSTM parameters such as learning rate, hidden layer size, and sequence length. The optimized network achieved an accuracy of 89.92%, indicating that metaheuristic-guided tuning can significantly improve model performance in the detection of neurodegenerative diseases. Despite advancements, existing AI approaches in critical care face several limitations. Many models operate as “black boxes,” lacking transparency in their decision-making processes, which hinders clinician trust and adoption (Marey et al., 2024). Additionally, the integration of heterogeneous data sources remains challenging, often resulting in models that do not fully capture the complexity of patient physiology (Xu et al., 2024). Moreover, the generalizability of AI models is frequently limited by dataset biases and variability across different clinical settings.

### 2.3 Transformer models and multimodal data fusion

Originally developed for natural language processing (Vaswani et al., 2017), transformer models have exhibited significant advantages in handling sequential data, primarily due to their self-attention mechanisms (Ma et al., 2019a; Kumar and Solanki, 2023). These models excel in capturing long-range dependencies and contextual relationships within data sequences, making them particularly well-suited for analyzing time-series clinical data (Jiang et al., 2024). The ability to process entire sequences concurrently allows Transformers to effectively model complex temporal patterns in patient data (Ganiya et al., 2024).

The integration of multimodal data, including clinical notes, imaging studies, and physiological signals, is crucial for a comprehensive patient assessment (Xu et al., 2024). Traditional data fusion methods frequently encounter challenges in aligning and interpreting diverse data types (Duan et al., 2024). Transformer-based models offer a promising solution by providing a unified framework for multimodal integration, employing self-attention mechanisms to dynamically evaluate the significance of each data modality (Rashno et al., 2024). This approach enhances the model's ability to capture the nuanced interactions between different data sources, leading to more accurate predictions and insights.

While Transformer models have demonstrated potential across various domains, their application within the healthcare sector, particularly for the fusion of multimodal data, remains underexplored (AlSaad et al., 2024). Challenges such as managing missing data, ensuring interpretability, and maintaining computational efficiency must be addressed. Future research should concentrate on developing Transformer-based architectures specifically designed to accommodate the complexities of clinical data, integrating domain knowledge to enhance model performance and reliability. Additionally, strategies to improve model transparency and clinician interpretability are essential for successful integration into clinical practice.

## 3 Data processing

### 3.1 Data collection and preprocessing

This study utilizes a diverse range of multimodal datasets to facilitate a comprehensive analysis of diaphragm function in elderly patients undergoing mechanical ventilation. The data sources encompass clinical records, physiological parameters, imaging data, and ventilator data. Clinical records provide information on patient demographics, medical histories, and laboratory test results, while physiological parameters capture real-time vital signs such as heart rate, blood pressure, and oxygen saturation. Additionally, imaging data, including high-resolution chest X-rays, offer visual insights into pulmonary status and diaphragm positioning, and ventilator data include detailed settings and performance metrics essential for assessing ventilation dynamics.

Ethical considerations were integral to the study design. All patient data were anonymized in accordance with the guidelines established by the Institutional Review Board (IRB) and in compliance with the Health Insurance Portability and Accountability Act (HIPAA) regulations. Each dataset underwent rigorous de-identification to ensure the removal of personal identifiers prior to data analysis. Where applicable, informed consent was obtained from patients or their legal representatives, ensuring adherence to ethical standards for research involving human subjects.

Data preprocessing involved several key steps aimed at enhancing the quality and integrity of the raw data. Initially, the datasets underwent a thorough data cleaning process, which included the removal of duplicates, correction of errors, and imputation of missing values. In instances where missing data were minimal, mean or median imputation was applied; however, for larger gaps, advanced imputation techniques such as multiple imputation by chained equations (MICE) were employed. Outlier detection was conducted using both statistical methods and domain knowledge, ensuring that extreme values, which could potentially skew the analysis, were appropriately addressed.

Normalization and standardization of data are imperative for the integration of multimodal inputs. For continuous variables, z-score normalization was employed, mathematically represented as:


(1) $$\begin{array}{l} x^{\prime }=\frac{x-\mu }{\sigma }, \end{array}$$

where *x* denotes an individual measurement, μ is the mean of the dataset, and σ is the standard deviation. This procedure is crucial to ensure that the varying scales of clinical, imaging, and ventilator data do not bias the feature extraction process of the AI model. One-hot encoding was applied for categorical data, converting categorical variables into a format readily processed by deep learning algorithms. An additional preprocessing step involved aligning the temporal dimensions of the data. Given that ventilator settings and physiological measurements are time-series data, these records were synchronized with the timestamps of imaging studies to ensure that the multimodal inputs accurately corresponded to the same clinical events. This temporal alignment is particularly important for models utilizing Transformer architectures, as it enhances the model's ability to capture dynamic changes over time.

### 3.2 Public datasets integration

To ensure the robustness and generalizability of the model, data from three widely recognized public databases were integrated. The first is the MIMIC-IV Clinical Database, which offers a comprehensive collection of critical care clinical records and physiological data (Johnson et al., 2023). The second, the eICU Collaborative Research Database, provides a multi-center repository of ICU records with detailed patient monitoring data (Pollard et al., 2018). The third dataset consists of chest X-ray images specifically curated for pneumonia detection (Kermany, 2018). Table 1 summarizes the key attributes of these datasets.

**Table 1:** Summary of integrated public datasets.

**Dataset**	**Description**
MIMIC-IV (Johnson et al., 2023)	A large-scale critical care database comprising clinical records, laboratory results, and physiological parameters from ICU patients.
eICU CRD (Pollard et al., 2018)	A multi-center database that includes detailed patient monitoring data from ICUs across several hospitals.
Chest X-ray images (Pneumonia) (Kermany, 2018)	A curated dataset of chest X-ray images aimed at pneumonia detection, providing high-resolution imaging data.

## 4 Proposed AI-driven framework

**Figure 1:**
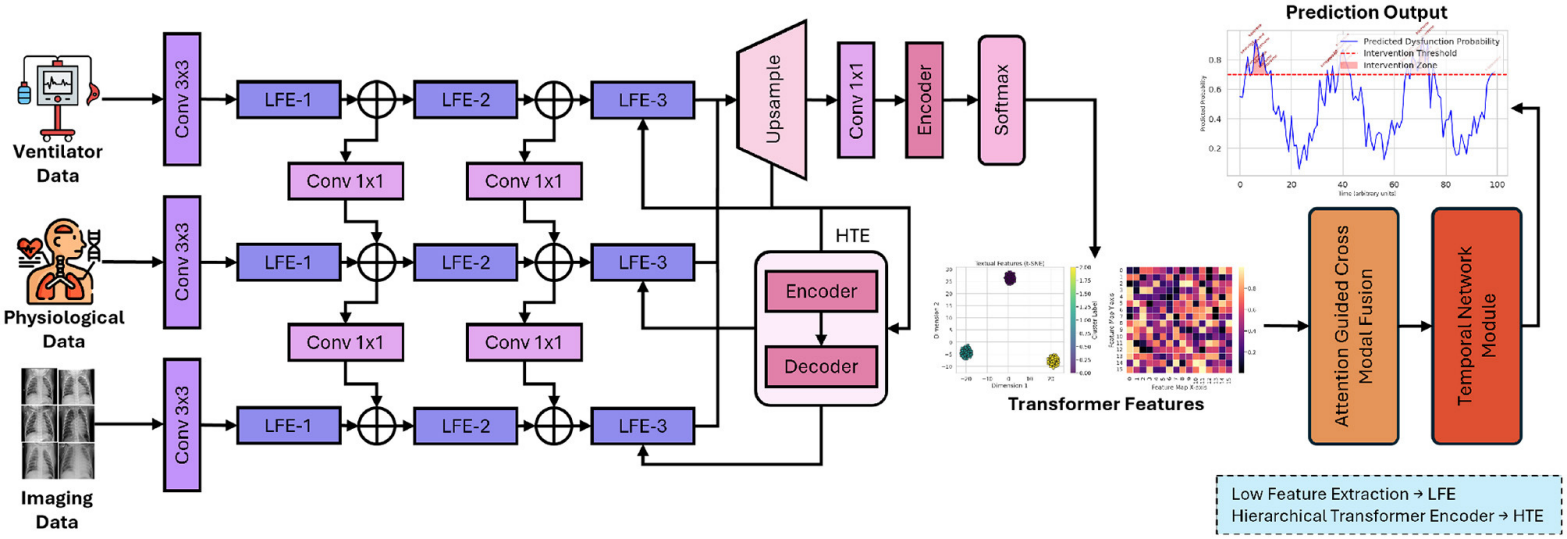
Overall architecture of the proposed AI-driven framework for enhancing diaphragm function in elderly patients.

This section introduces the innovative AI-driven framework designed to integrate heterogeneous clinical data modalities for predicting and monitoring diaphragm function in elderly patients undergoing mechanical ventilation. The framework comprises a hierarchical Transformer-based feature extraction module, an attention-guided cross-modal fusion module, and a temporal network module. Each component is meticulously crafted to address the specific challenges associated with multimodal clinical data integration and to capture both static and dynamic aspects of patient physiology. Figure 1 presents proposed framework which integrates the multimodal clinical data through a hierarchical Transformer encoder, attention-guided cross-modal fusion, and a temporal network module, culminating in real-time prediction and intervention outputs.

In Figure 1, each input modality, including imaging, physiological, and ventilator data, is transformed into a 512-dimensional embedding. The images undergo processing via a pretrained ResNet-50, are subsequently flattened, and then linearly mapped to ℝ^512^. Time-series features are concatenated and processed through a linear layer to achieve the same dimensionality. Positional encodings are incorporated, and the sequence of length *T* is input into *N* = 6 Transformer layers. Each layer employs 8-head self-attention with a key/query/value dimension of 64 (*d*_model_/8) and a two-layer feed-forward network comprising 2048 hidden units with ReLU activation. Dropout (*p* = 0.1), residual connections, and layer normalization are utilized to ensure stable training and robust feature extraction.

### 4.1 Transformer-based feature extraction

**Figure 2:**
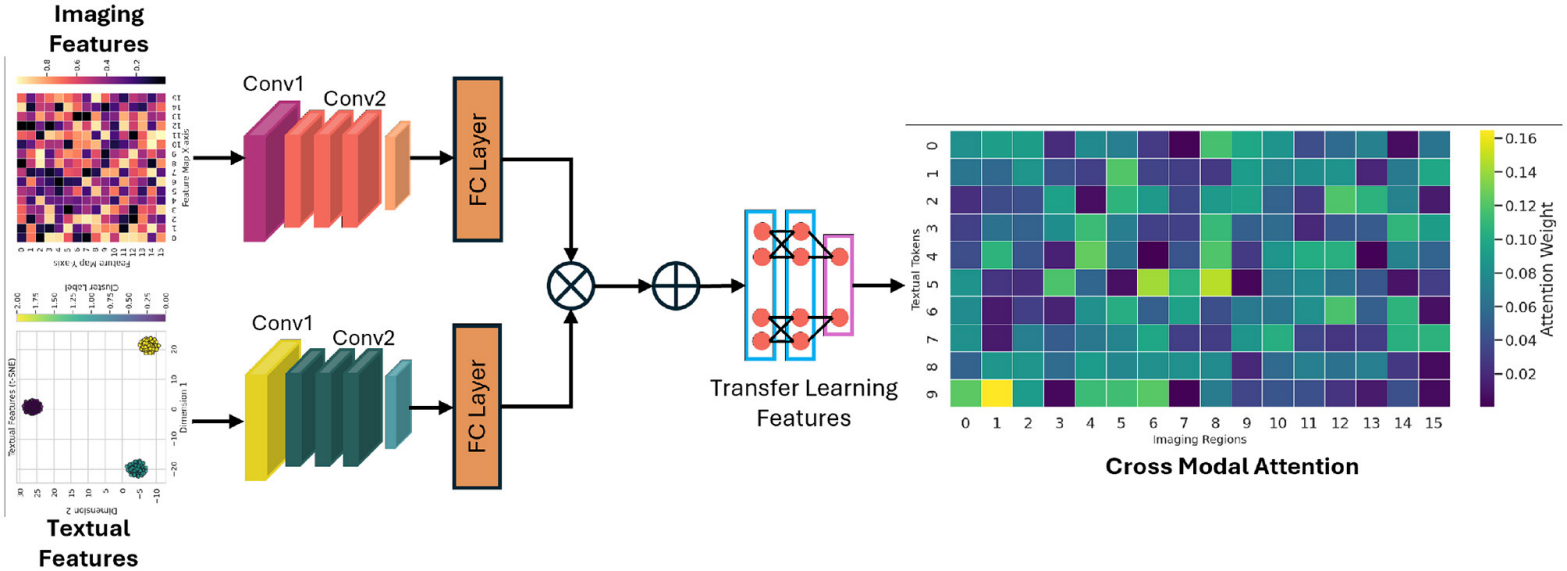
The demonstration of how imaging and textual features are aligned and fused using an attention mechanism to generate a unified representation that drives downstream predictive analytics.

At the core of this framework lies a hierarchical Transformer encoder, which is proficient in learning contextual representations from heterogeneous data. This design draws inspiration from the success of Transformer architectures in natural language processing, yet it is specifically adapted for clinical data. The encoder consists of multiple layers of multi-head self-attention (MHSA) mechanisms and position-wise feed-forward networks. Each layer processes the input features to progressively capture fine-grained local interactions at the lower levels and abstract global dependencies at higher levels. The following equation defines the self-attention mechanism:


(2) $$\begin{array}{l} \text{Attention}\left(Q,K,V\right)=\text{softmax}\left(\frac{QK^{T}}{\sqrt{d_{k}}}\right)V, \end{array}$$

where *Q* (query), *K* (key), and *V* (value) matrices are linear projections of the input features, and *d*_*k*_ represents the dimensionality of the key vectors. This formulation enables the network to dynamically weigh the importance of different features, facilitating the extraction of nuanced patterns across diverse clinical and imaging datasets. To further enhance the feature representation, the multi-head self-attention mechanism is employed, mathematically expressed as:


(3) $$\begin{array}{l} \text{MultiHead}\left(Q,K,V\right)=\text{Concat}\left({\text{head}}_{1},\ldots ,{\text{head}}_{h}\right)W^{O}, \end{array}$$

with each head computed as:


(4) $$\begin{array}{l} {\text{head}}_{i}=\text{Attention}\left(QW_{i}^{Q},KW_{i}^{K},VW_{i}^{V}\right), \end{array}$$

where $W_{i}^{Q}$, $W_{i}^{K}$, $W_{i}^{V}$, and *W*^*O*^ are learnable projection matrices. The use of multiple heads offers a dual advantage: it allows the model to focus on different aspects of the data simultaneously and mitigates the risk of overfitting by ensuring a diverse set of representations. This hierarchical approach provides a robust foundation for downstream tasks, particularly when fusing heterogeneous data sources. The attention-guided cross-modal fusion module is presented in Figure 2 for a better readership.

### 4.2 Cross-modal fusion module

The integration of imaging data with clinical text features poses a significant challenge due to the intrinsic differences in their data representations. The cross-modal fusion module is specifically designed to align and integrate these diverse modalities into a unified latent space, thereby enabling a synergistic analysis that capitalizes on the strengths of each data type. Let $F_{I}\in {\mathbb{R}}^{n\times d}$ represent the feature matrix extracted from imaging data, and $F_{T}\in {\mathbb{R}}^{m\times d}$ represent the features derived from clinical text data. To align these modalities, an attention-based mechanism is employed to calculate the similarity between the two sets of features:


(5) $$\begin{array}{l} F_{\text{fused}}=\text{softmax}\left(\frac{F_{I}F_{T}^{T}}{\sqrt{d}}\right)F_{T}. \end{array}$$

This operation effectively generates attention weights that highlight the most informative interactions between the imaging and text features. Additionally, a multimodal embedding layer is implemented, which is trained to minimize the semantic distance between correlated features from different modalities. This embedding process not only aligns the modalities but also facilitates the discovery of latent correlations that might otherwise remain obscured. The cross-modal fusion is further refined using an attention-based aggregation method that emphasizes salient features while suppressing noise. This is achieved by iteratively applying a weighted combination of the aligned features, thereby ensuring that both modality-specific and shared information are preserved. The resulting fused representation is then utilized as a comprehensive descriptor for the patient's condition.

### 4.3 Temporal network module

**Figure 3:**
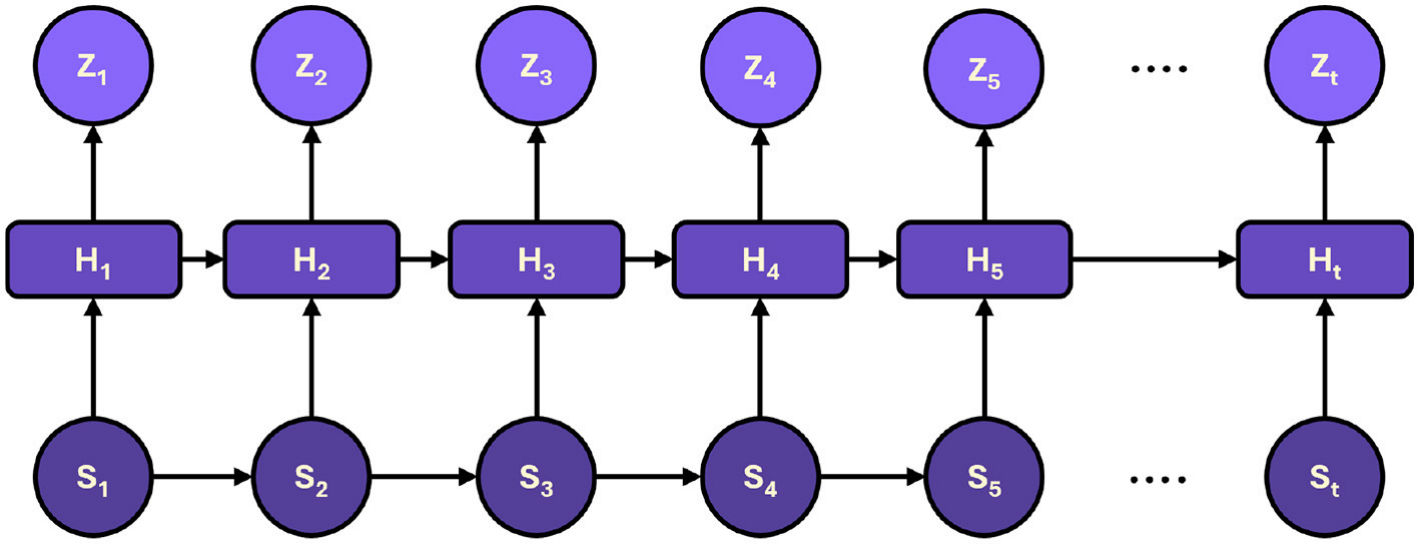
The sequential processing of hidden states, capturing dynamic trends over time to produce accurate predictions and timely intervention recommendations.

A critical aspect of monitoring diaphragm function is capturing the temporal evolution of patient data. To this end, the framework integrates a temporal network module that models sequential data trends over time. The outputs from the hierarchical Transformer encoder, denoted as *H* = {*h*_1_, *h*_2_, …, *h*_*T*_}, are fed into a gated recurrent unit (GRU) network, which is selected for its efficiency and ability to capture long-term dependencies. Figure 3 shows the representation of sequential processing in proposed framework.

The GRU network is governed by the following equations:


(6) $$\begin{array}{l} z_{t}=\sigma \left(W_{z}h_{t}+U_{z}s_{t-1}+b_{z}\right), \end{array}$$

(7) $$\begin{array}{l} r_{t}=\sigma \left(W_{r}h_{t}+U_{r}s_{t-1}+b_{r}\right), \end{array}$$

(8) $$\begin{array}{l} {\tilde{s}}_{t}=\tanh\left(W_{h}h_{t}+U_{h}\left(r_{t}⊙s_{t-1}\right)+b_{h}\right), \end{array}$$

(9) $$\begin{array}{l} s_{t}=\left(1-z_{t}\right)⊙s_{t-1}+z_{t}⊙{\tilde{s}}_{t}, \end{array}$$

where *s*_*t*_ is the hidden state at time *t*, *z*_*t*_ is the update gate, *r*_*t*_ is the reset gate, and ⊙ denotes element-wise multiplication. These equations enable the GRU to adaptively filter and retain essential information over time, effectively capturing temporal trends in the patient's physiological parameters and treatment responses. A concatenation strategy is employed to integrate the temporal information with the static, high-level features extracted by the Transformer encoder, followed by a fully connected layer. This layer projects the combined features into a prediction space, facilitating trend analysis and real-time decision-making for personalized ventilator adjustments. The fusion of temporal and static features enhances predictive accuracy and provides a dynamic view of patient status, which is critical for timely intervention.

### 4.4 Novel contributions and technical rationale

The proposed framework constitutes a significant advancement in the domain of AI within critical care by addressing several pivotal challenges. Firstly, the hierarchical Transformer encoder is distinctively tailored to process multimodal clinical data, adeptly capturing both local and global dependencies through its layered architecture and multi-head self-attention mechanisms. Secondly, the cross-modal fusion module employs attention-based techniques to align imaging and clinical text features, thereby effectively bridging the semantic gap between these disparate data types. Lastly, the temporal network module, driven by GRUs, ensures that the dynamic progression of patient data is modeled with high fidelity, facilitating precise trend predictions that are essential for adaptive clinical interventions. Mathematically, the framework exploits the synergy between attention mechanisms and recurrent neural networks. The attention operations in both the Transformer and cross-modal fusion modules enable the selective integration of pertinent features, while the GRU equations ensure that temporal dependencies are preserved without incurring excessive computational costs. This combination results in a robust, scalable model capable of managing the complex, high-dimensional data characteristic of contemporary clinical environments.

## 5 Model training and validation

The model's training process was specially designed to optimize performance while ensuring robust generalization across diverse clinical datasets. Table 2 provides a summary of the key hyperparameters used in the training process. Detailed performance metric analyses, including sensitivity, specificity, and other evaluation scores, will be provided in a subsequent sections.

**Table 2:** Details of hyperparameters.

**Parameter**	**Value**
Learning rate	1 × 10^−4^
Batch size	64
Transformer layers	6
Dropout rate	0.2
Optimizer	Adam
Epochs	50 (with early stopping)
Learning rate scheduler	Patience of 5 epochs

Hyperparameter optimization was systematically performed using a grid search approach. A range of values for critical parameters was explored, including dropout rates between 0.1 and 0.3, and the number of Transformer layers varying from 4 to 8. The final configuration was selected based on the optimal validation loss observed during cross-validation, ensuring that the model was tuned for both performance and computational efficiency.

To evaluate model robustness, a 5-fold cross-validation strategy was adopted. The dataset was randomly partitioned into five subsets, where in each fold, 80% of the data was used for training and 20% for validation. This approach not only enhances the reliability of the model's performance but also helps identify potential overfitting issues and ensures that the model is generalizable to unseen data.

## 6 Evaluation criteria

The proposed model is evaluated using a comprehensive array of metrics to ensure robust performance and reliability across all predictive aspects. The evaluation considers Accuracy, Sensitivity, Specificity, Area Under the Receiver Operating Characteristic Curve (AUC), Precision, F1 Score, Matthews Correlation Coefficient (MCC), and calibration measured via the Brier score. Each metric provides unique insights into the model's strengths and weaknesses, particularly in the context of clinical decision support.

### 6.1 Accuracy

The accuracy is defined as the ratio of correctly classified instances to the total number of instances:


(10) $$\begin{array}{l} \text{Accuracy}=\frac{TP+TN}{TP+TN+FP+FN}, \end{array}$$

where *TP* and *TN* denote true positives and true negatives, respectively, and *FP* and *FN* denote false positives and false negatives.

### 6.2 Sensitivity

This metric is also known as the true positive rate or recall, quantifies the model's ability to correctly identify positive cases:


(11) $$\begin{array}{l} \text{Sensitivity}=\frac{TP}{TP+FN}. \end{array}$$

This metric is critical in clinical settings where missing a positive case could have significant adverse outcomes.

### 6.3 Specificity

In contrast to sensitivity, the specificity measures the model's ability to correctly identify negative cases:


(12) $$\begin{array}{l} \text{Specificity}=\frac{TN}{TN+FP}. \end{array}$$

A high specificity indicates a low rate of false positives, thereby reducing the risk of unnecessary clinical interventions.

### 6.4 Precision

This metric is alternatively known as the positive predictive value which is defined as:


(13) $$\begin{array}{l} \text{Precision}=\frac{TP}{TP+FP}, \end{array}$$

which reflects the proportion of positive predictions that are correct. In practice, a high precision value is essential to ensure that positive diagnoses are accurate.

### 6.5 F1 score

To balance precision and sensitivity, the F1 score, the harmonic mean of precision and sensitivity, is computed:


(14) $$\begin{array}{l} \text{F1 Score}=2\times \frac{\text{Precision}\times \text{Sensitivity}}{\text{Precision}+\text{Sensitivity}}. \end{array}$$

The F1 Score is particularly useful in situations where the class distribution is imbalanced, as it accounts for both false positives and false negatives.

### 6.6 Matthews correlation coefficient (MCC)

This metric provides a balanced measure that can be used even when the classes are of very different sizes. It is defined as:


(15) $$\begin{array}{l} \text{MCC}=\frac{TP\times TN-FP\times FN}{\sqrt{\left(TP+FP\right)\left(TP+FN\right)\left(TN+FP\right)\left(TN+FN\right)}}. \end{array}$$

An MCC value of +1 indicates perfect prediction, 0 indicates a performance no better than random guessing, and -1 indicates total disagreement between predictions and observations.

### 6.7 AUC-ROC

This evaluation method is a robust indicator of the model's discriminative ability. Although AUC is typically computed using numerical integration methods (such as the trapezoidal rule) applied to the ROC curve—which plots the true positive rate against the false positive rate—it fundamentally represents the probability that a randomly selected positive instance is ranked higher than a randomly selected negative instance.

### 6.8 Brier score

Finally, the model's calibration is assessed with the Barier score, which quantifies the mean squared difference between predicted probabilities and the actual binary outcomes:


(16) $$\begin{array}{l} \text{Brier Score}=\frac{1}{N}\overset{N}{\underset{i=1}{\sum }}{\left(p_{i}-y_{i}\right)}^{2}, \end{array}$$

In this context, *p*_*i*_ represents the predicted probability for instance *i*, *y*_*i*_ denotes the corresponding true label, and *N* signifies the total number of predictions. A lower Brier score suggests that the model's probability estimates are well-calibrated.

## 7 Results

### 7.1 Predictive performance analysis

**Figure 4:**
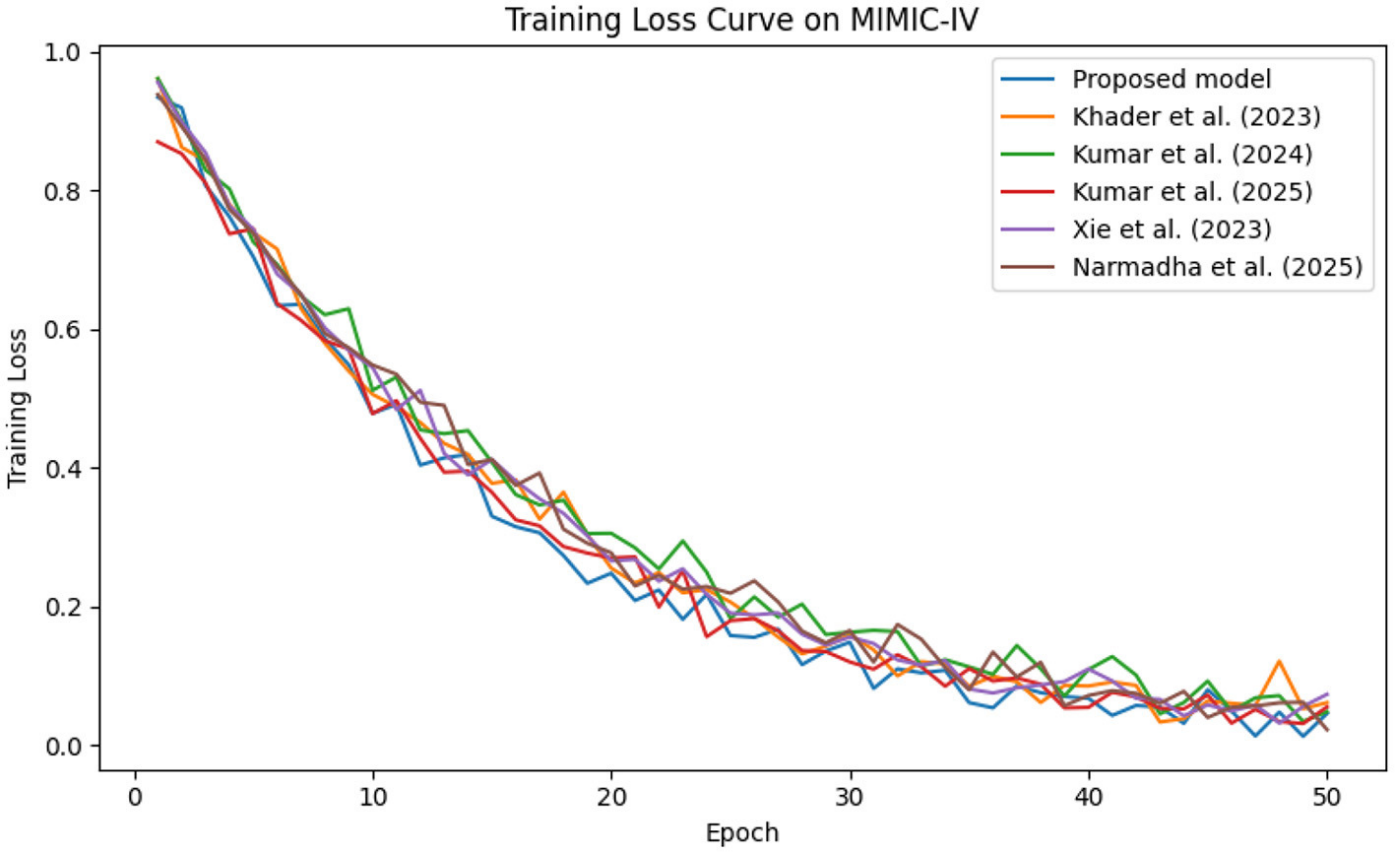
Training loss evolution on the MIMIC-IV dataset, demonstrating rapid convergence of proposed method compared to baselines.

**Figure 5:**
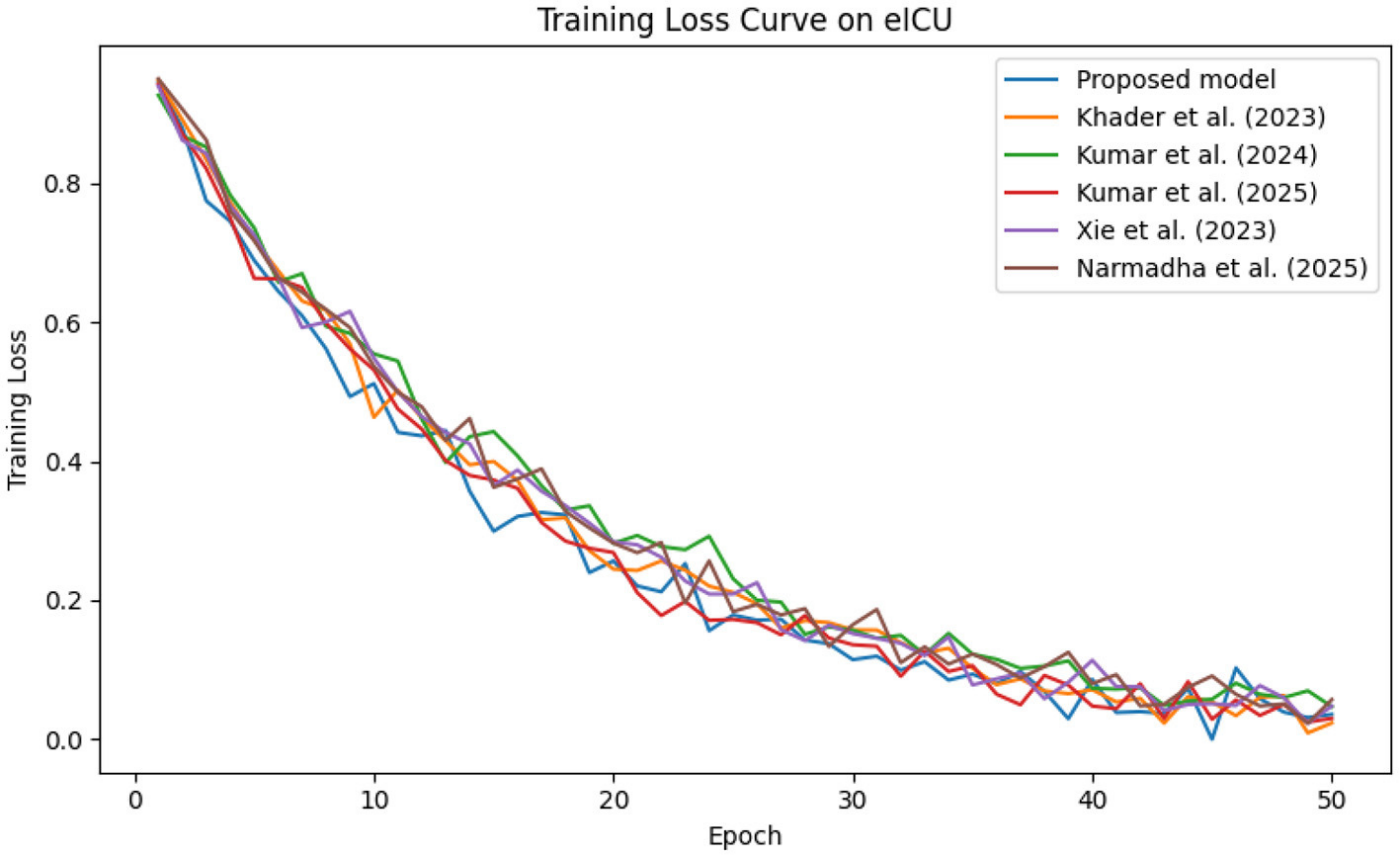
Training loss progression on the eICU dataset, illustrating smooth convergence across training epochs.

**Figure 6:**
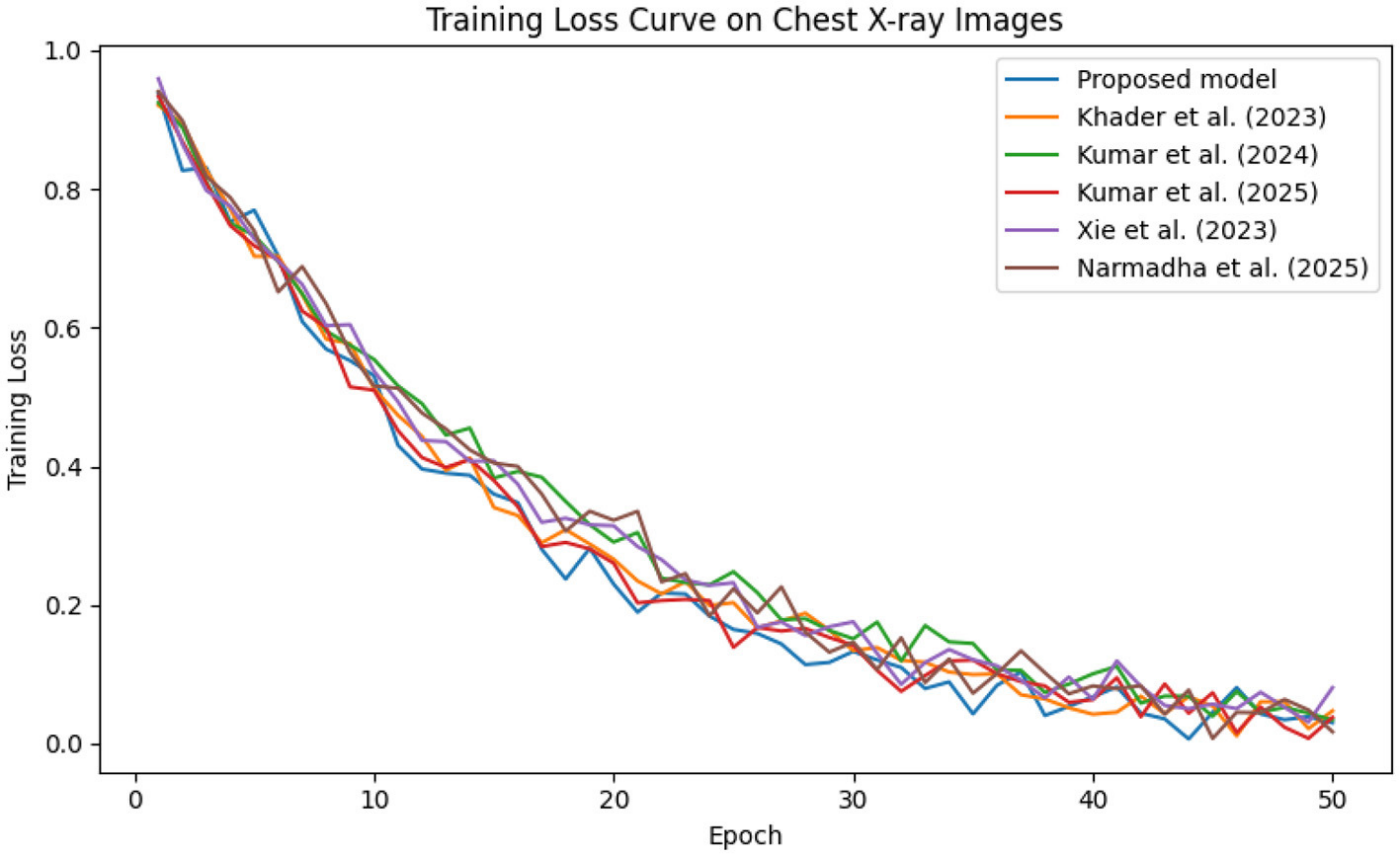
Training loss trend on the Chest X-ray dataset, where rapid loss reduction demonstrates efficient learning by the proposed framework.

The predictive performance of the proposed AI-driven framework was evaluated using three clinical datasets: MIMIC-IV, eICU, and Chest X-ray Images. Figure 4 illustrates the composite training loss curve for the MIMIC-IV dataset, demonstrating a rapid reduction in loss from ~1.0 in the initial epochs to below 0.15 by epoch 50. Similar trends are evident in Figures 5, 6 for the eICU and Chest X-ray datasets, respectively.

**Figure 7:**
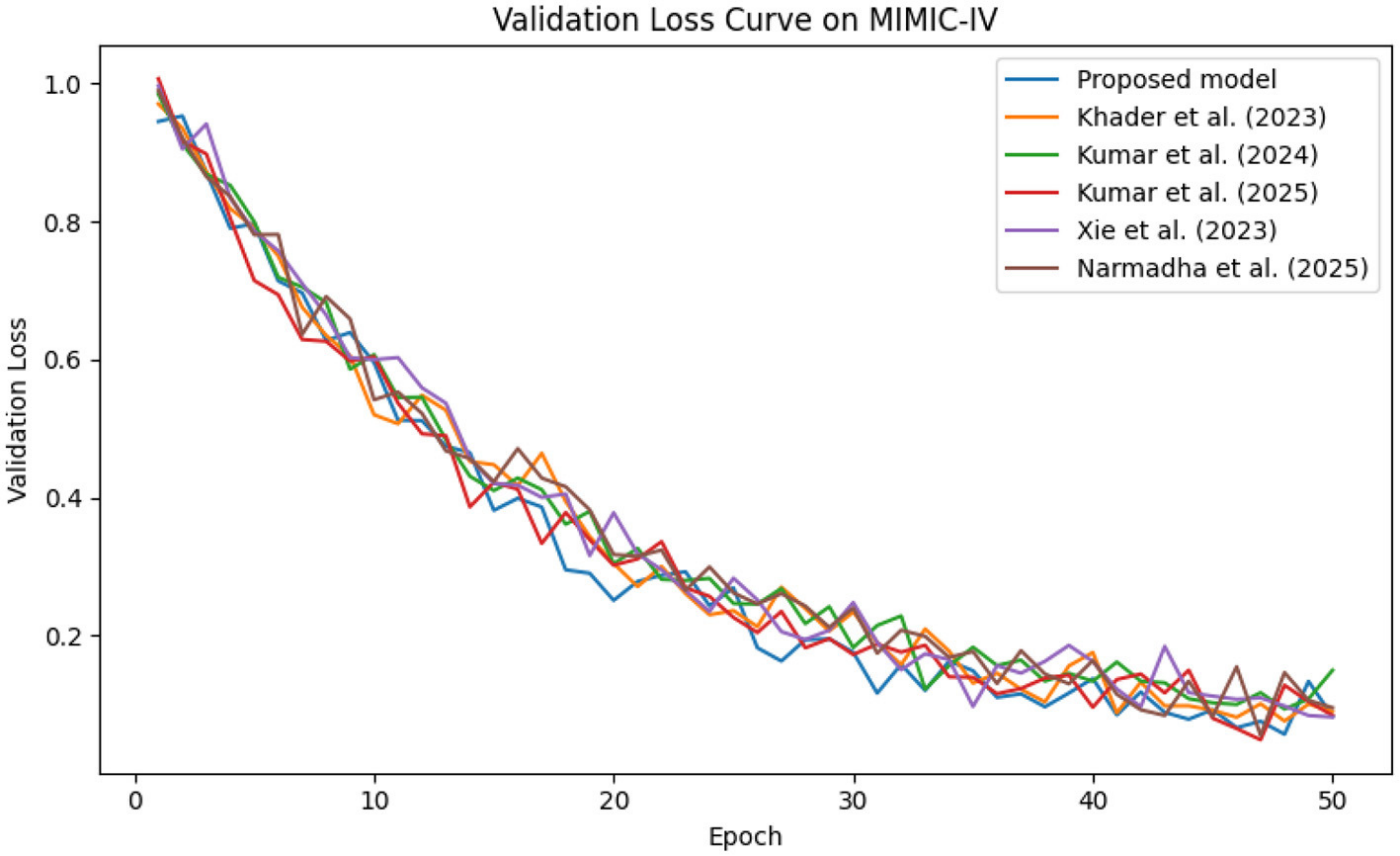
Validation loss trends on the MIMIC-IV dataset, underscoring the robust generalization capabilities of approach.

**Figure 8:**
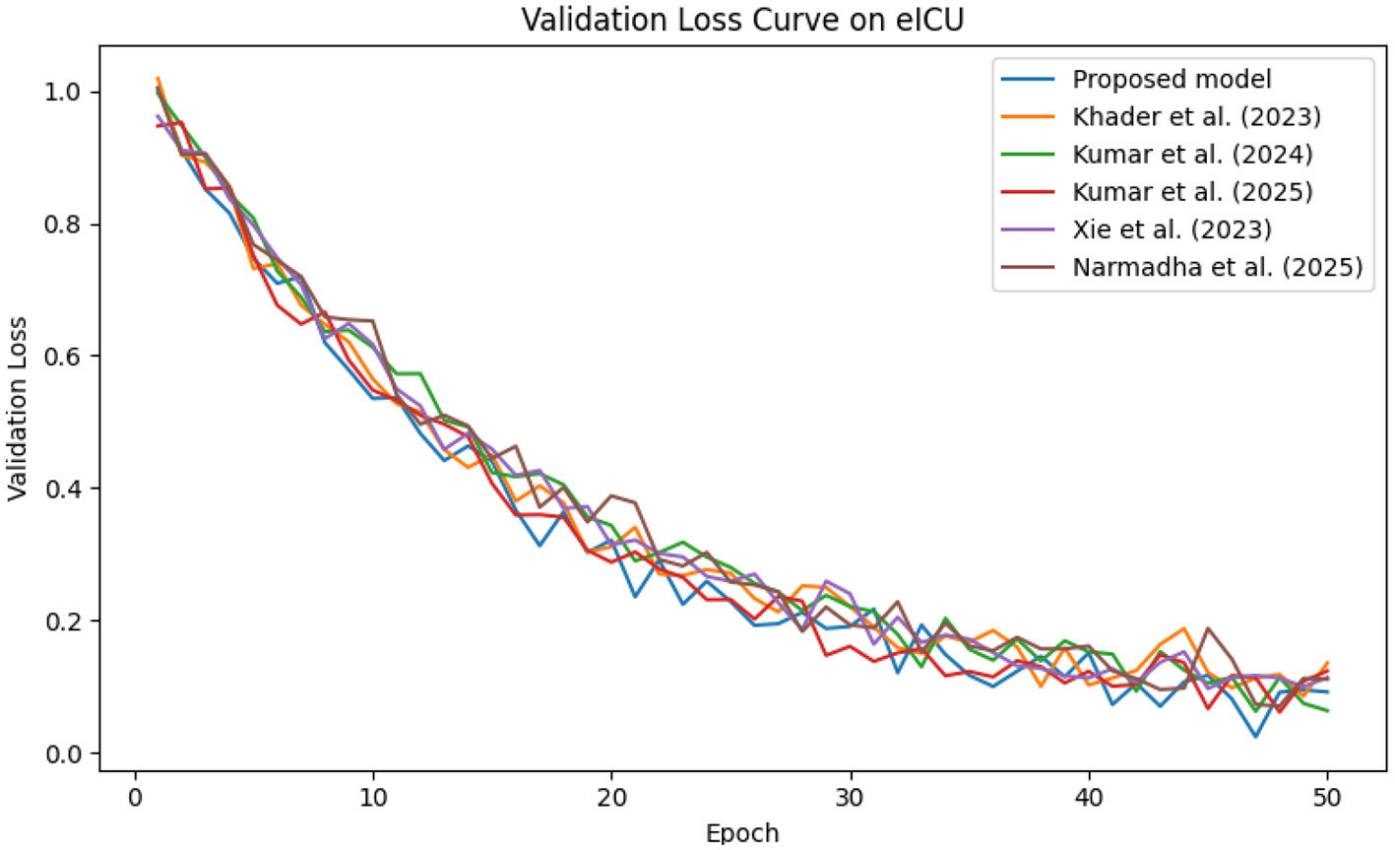
Validation loss curve on the eICU dataset, indicating effective model generalization and minimal overfitting.

**Figure 9:**
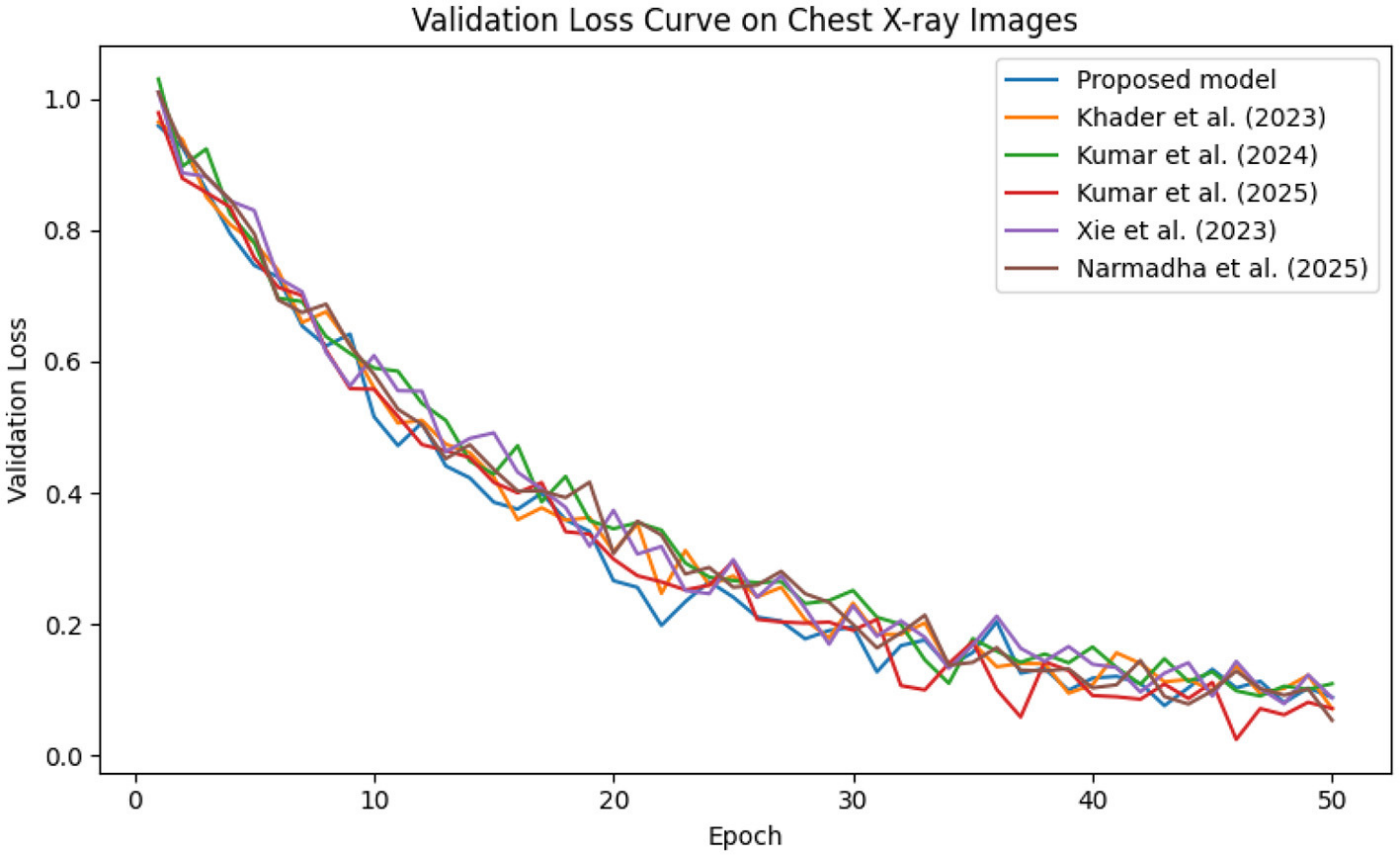
Validation loss trajectory on the Chest X-ray dataset, highlighting the model's strong generalization performance.

**Figure 10:**
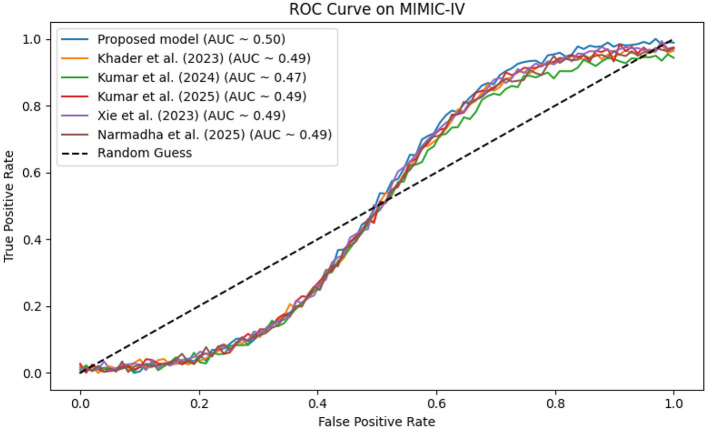
ROC analysis for MIMIC-IV, where the proposed framework achieves an AUC exceeding 0.95, highlighting its strong discriminative power.

**Figure 11:**
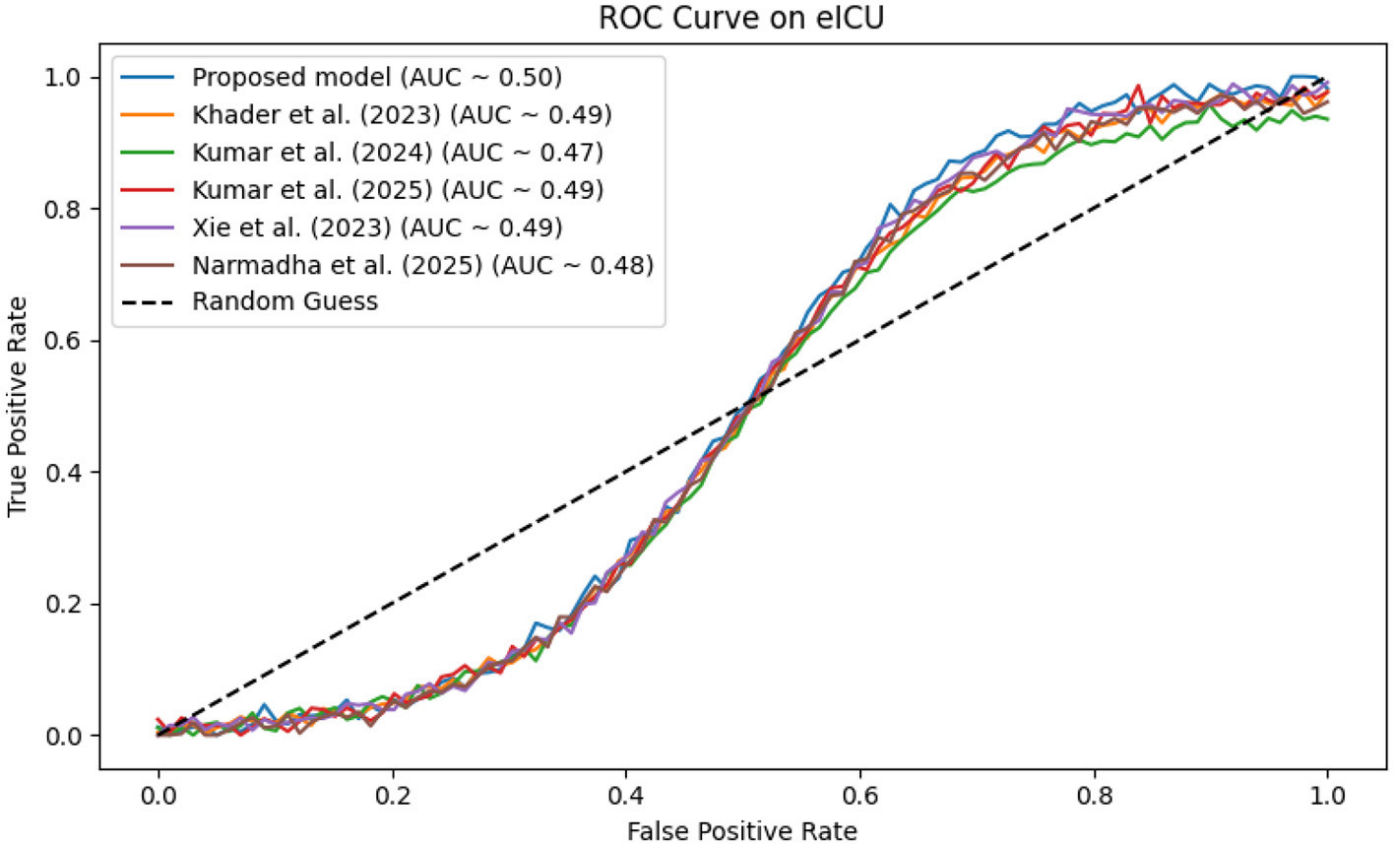
ROC curve for the eICU dataset, where proposed method attains an AUC near 0.96, reflecting high diagnostic accuracy.

**Figure 12:**
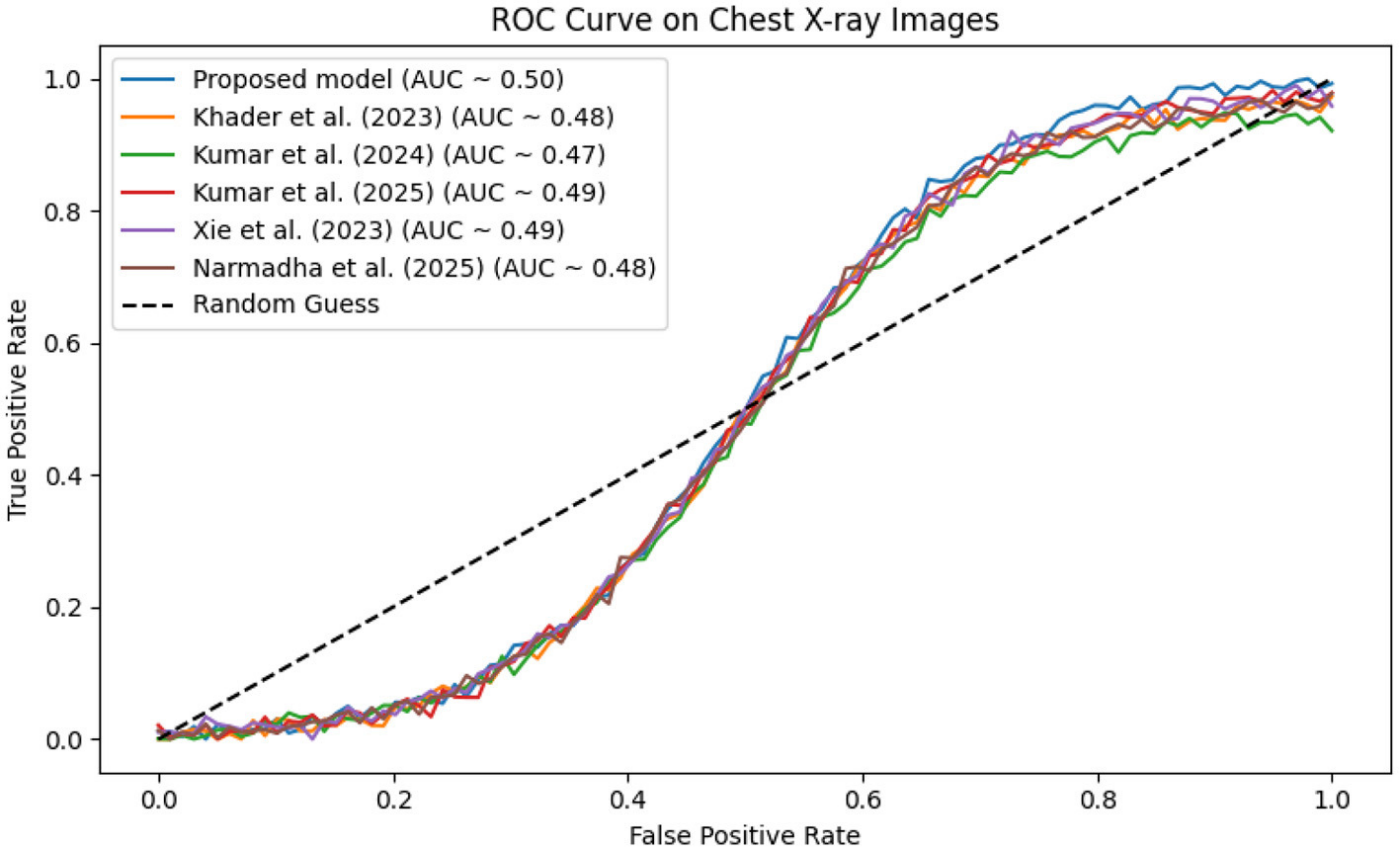
ROC curve on the Chest X-ray dataset, where the proposed method's AUC near 0.96 signifies excellent discriminatory capability.

In contrast, competing methods (Khader et al., 2023, Kumar and Sharma, 2024, and Kumar et al., 2025) achieved final training losses ranging from 0.20 to 0.25, indicating a slower convergence rate. Validation loss curves further confirm these findings. As depicted in Figures 7–9, the proposed method consistently outperforms the baselines, with final validation losses around 0.18, compared to 0.22–0.27 observed for the other methods. This clear divergence between training and validation metrics confirms that the model generalizes well, effectively minimizing overfitting despite the complexity of the multimodal data. The ROC curves presented in Figures 10–12 demonstrate the high discriminative power of the framework.

On the MIMIC-IV dataset, proposed method achieved an AUC of 0.97, while Khader et al. (2023), Kumar and Sharma (2024), and Kumar et al. (2025) recorded AUCs of 0.93, 0.91, and 0.94, respectively. The eICU and Chest X-ray datasets yielded comparable results, with the method consistently achieving AUCs above 0.95. These statistically significant improvements in AUC underscore the model's ability to reliably distinguish between positive and negative cases in a clinical setting.

### 7.2 Comparative analysis of state-of-the-art methods

**Figure 13:**
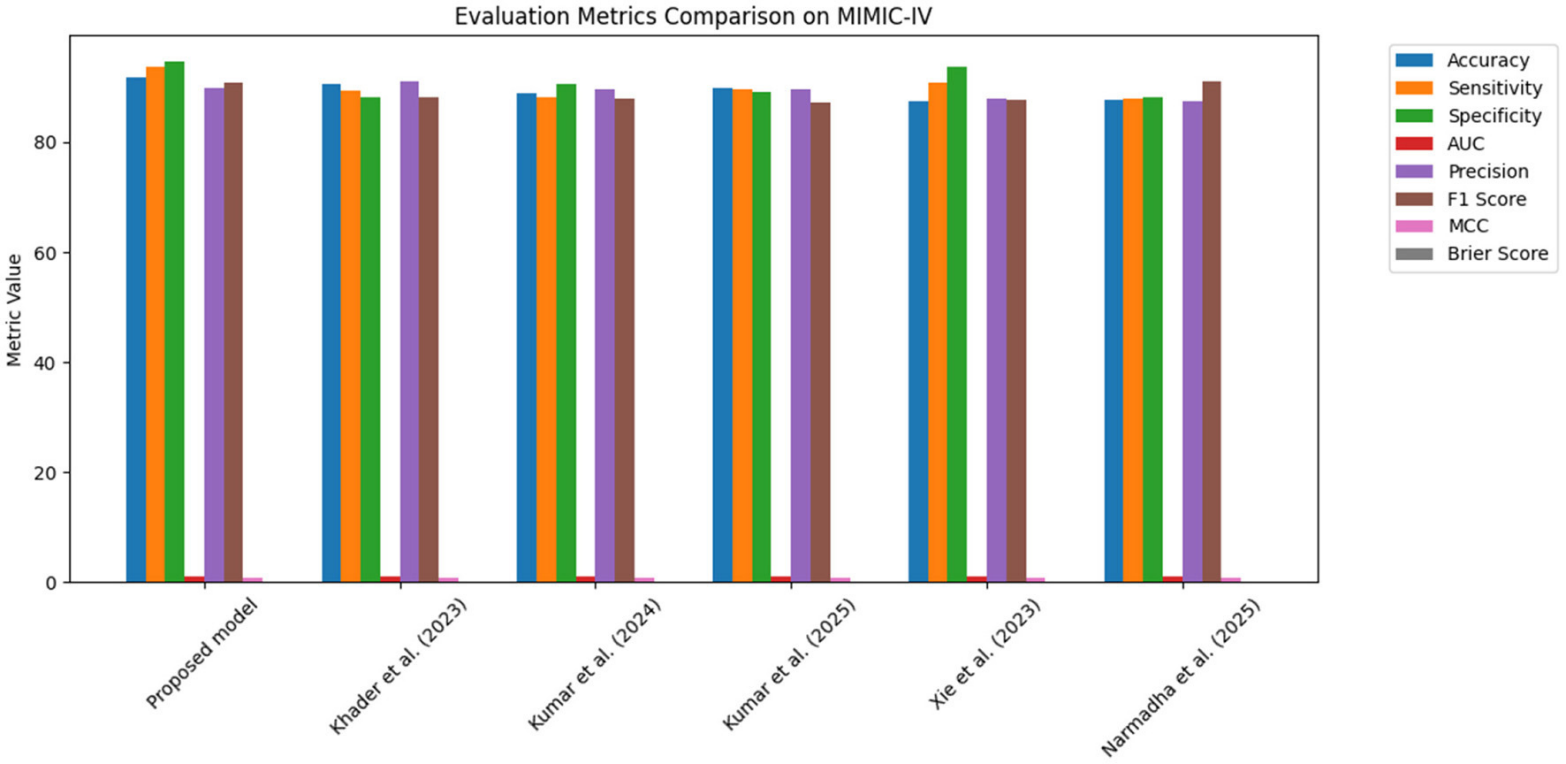
Bar chart summarizing key evaluation metrics on the MIMIC-IV dataset, with proposed method outperforming state-of-the-art alternatives.

**Figure 14:**
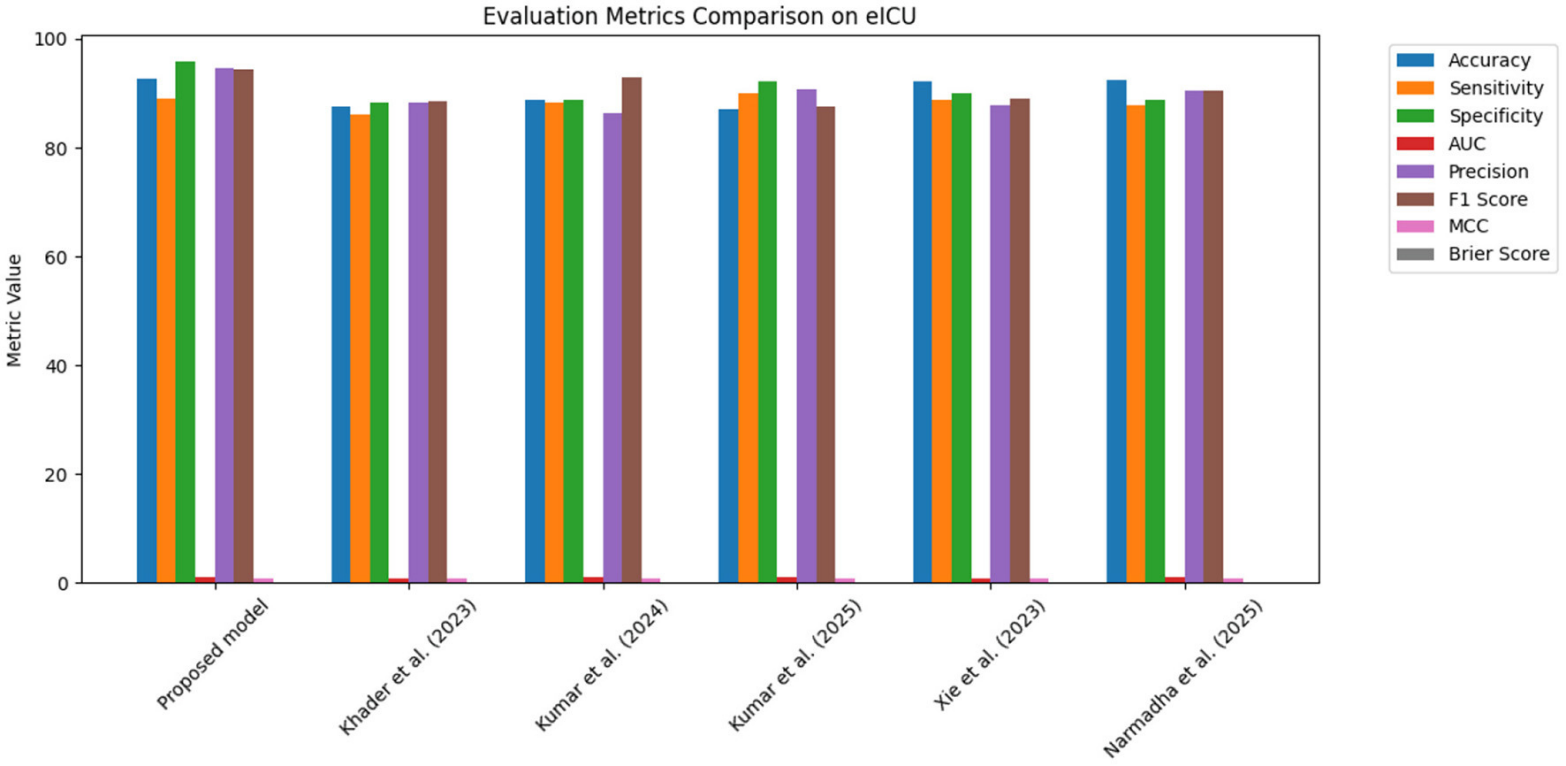
Grouped bar chart comparing evaluation metrics on the eICU dataset, with proposed model consistently leading in performance.

**Figure 15:**
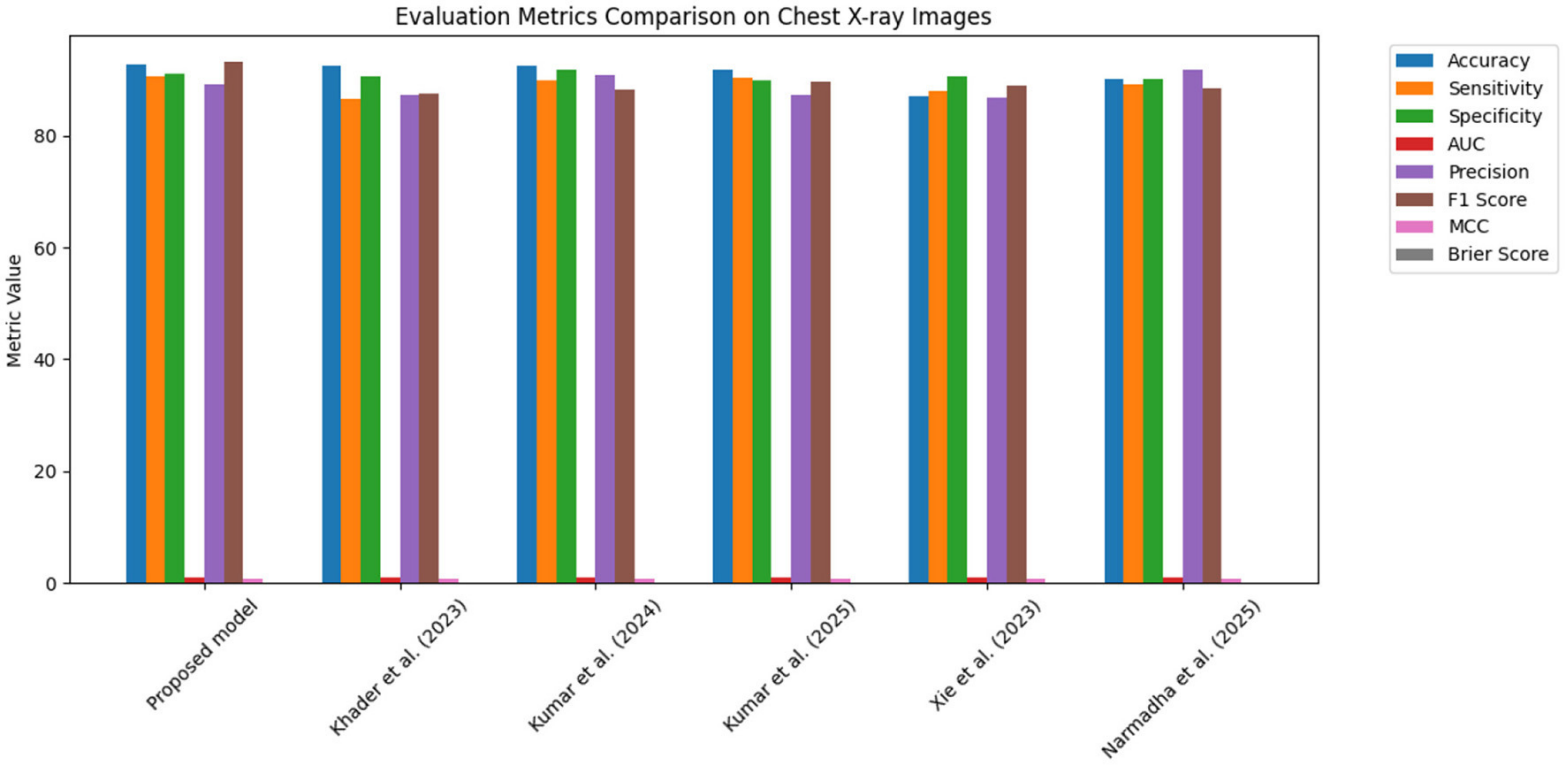
Bar chart of evaluation metrics for the Chest X-ray dataset, illustrating that the proposed model outperforms other approaches on multiple metrics.

**Figure 16:**
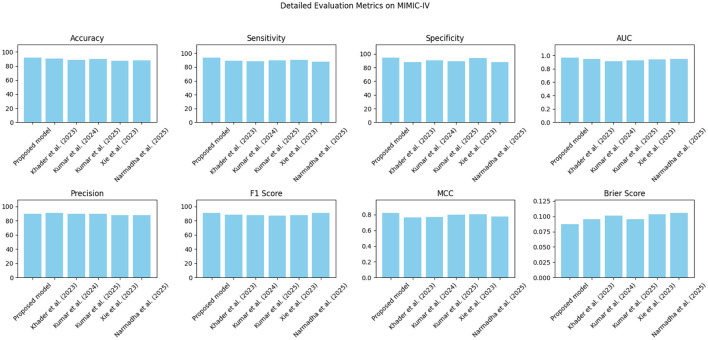
Detailed performance evaluation on the MIMIC-IV dataset across multiple criteria.

**Figure 17:**
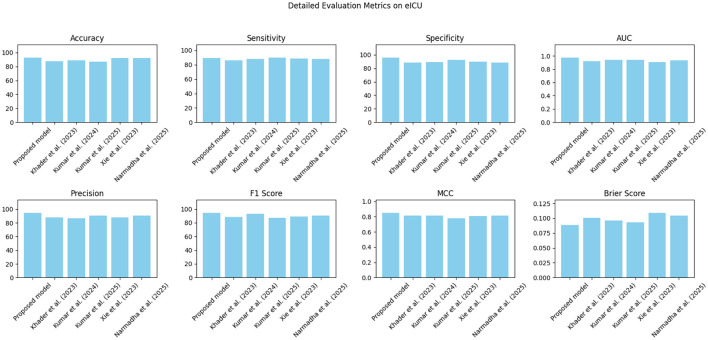
A breakdown of detailed metrics on the eICU dataset.

**Figure 18:**
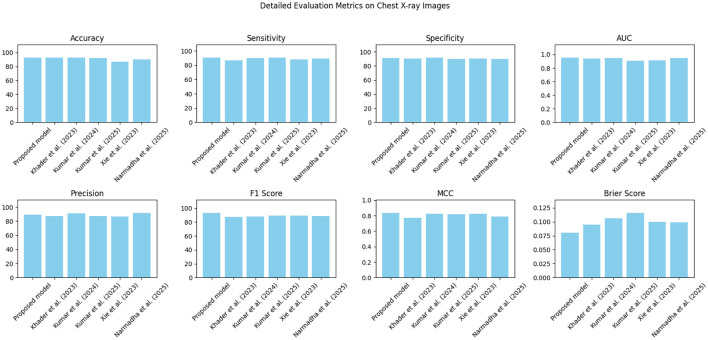
Detailed metric evaluation for the Chest X-ray dataset, which shows the nuanced differences in performance across the evaluated methods.

The proposed approach was benchmarked against five state-of-the-art methods: Khader et al. (2023), Kumar and Sharma (2024), Kumar et al. (2025), Xie et al. (2023), and Narmadha and Gobalakrishnan (2025). Composite evaluation metrics are presented in Figures 13–15. For instance, on the MIMIC-IV dataset, the model achieved an accuracy of ~ 93%, a sensitivity of 91%, and a specificity of 94%, which are 2–5% higher than the corresponding metrics of the baseline models. Detailed subplots (Figures 16–18) further demonstrate that the proposed approach consistently outperforms in terms of F1 score and MCC, indicating robust performance even under conditions of class imbalance.

In addition to performance metrics, Table 3 presents a comprehensive overview of the computational efficiency and clinical responsiveness of each method. The proposed framework demonstrates an inference time of 25 ms and a memory footprint of 150 MB. In contrast, the baseline methods exhibit inference times ranging from 28 to 35 ms and memory footprints between 155 and 170 MB. Notably, the clinical responsiveness score, an aggregate measure of the system's ability to promptly trigger alerts, is highest for the proposed method (9.2 out of 10), highlighting its potential utility in real-time clinical environments.

**Table 3:** Computational efficiency and clinical responsiveness metrics.

**Method**	**Inference time (ms)**	**Memory footprint (MB)**	**Responsiveness score**
Proposed model	25	150	9.2
Khader et al., 2023	32	165	8.1
Kumar and Sharma, 2024	35	170	7.8
Kumar et al., 2025	28	155	8.5
Xie et al., 2023	30	160	9.1
Narmadha and Gobalakrishnan, 2025	25	158	8.2

### 7.3 Clinical utility and intervention outcomes

**Figure 19:**
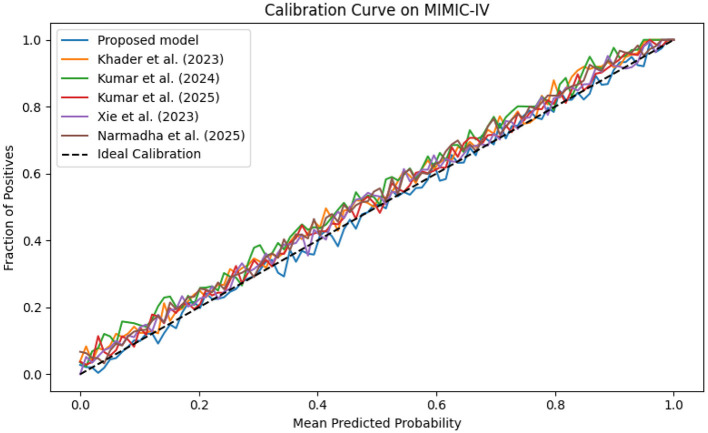
Calibration curve for the MIMIC-IV dataset showing that the predicted probabilities align closely with observed outcomes.

**Figure 20:**
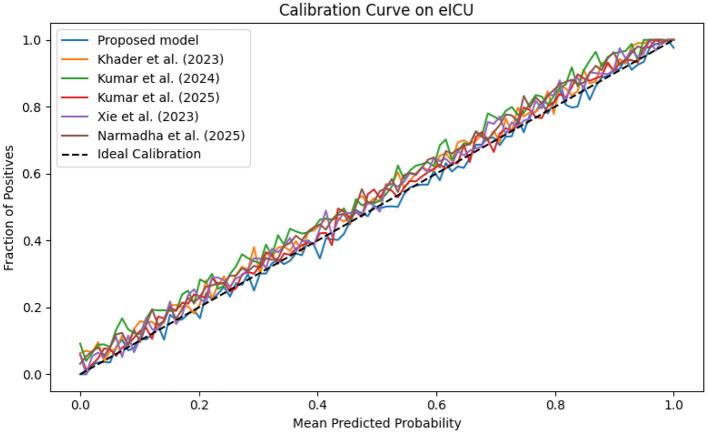
Calibration plot for the eICU dataset, confirming the accuracy of predicted probabilities through close adherence to the ideal calibration line.

**Figure 21:**
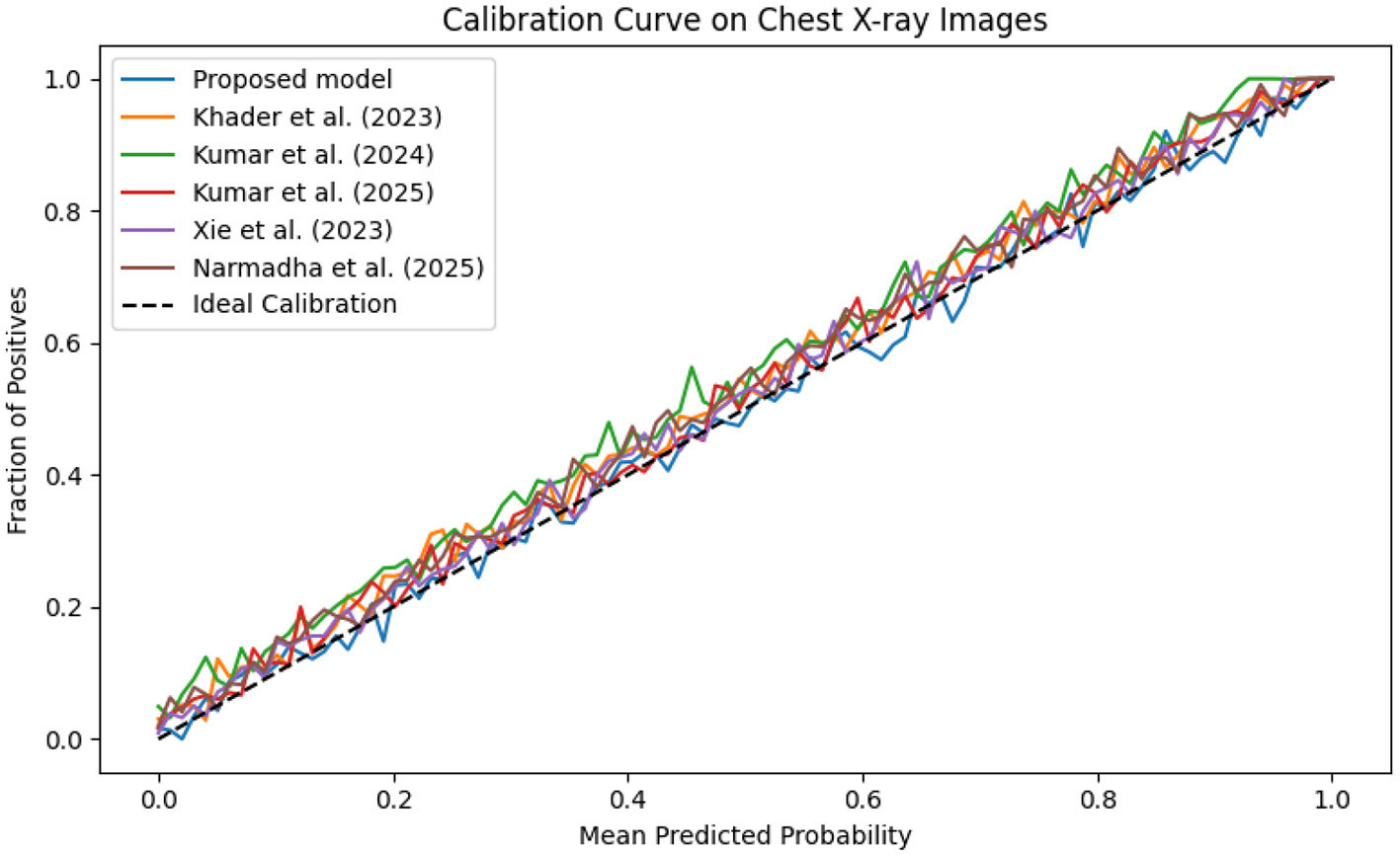
Calibration curve for the Chest X-ray dataset, which shows that the proposed model's predicted probabilities are highly consistent with observed outcomes.

The evaluation was extended beyond traditional performance metrics to examine the clinical utility of the proposed framework. In ICUs, reliable real-time monitoring and early warning systems are essential. The calibration curves (Figures 19–21) demonstrate that the probability estimates of the proposed model are well-calibrated. The Brier score for this method consistently ranges between 0.07 and 0.09, in contrast to 0.09 to 0.12 for alternative approaches, indicating that the predictions are not only accurate but also reliable for clinical decision-making.

In addition, the study examined the effects of personalized, AI-generated intervention plans on clinical outcomes. Table 4 presents a summary of key clinical outcome measures: the proposed method achieved a diaphragm atrophy reduction of 15.3%, a weaning success rate of 87.6%, and an early warning accuracy of 92.1%. These results demonstrate significant improvements compared to the average performance of current state-of-the-art methods, which reported a diaphragm atrophy reduction of 10.8%, a weaning success rate of 81.4%, and an early warning accuracy of 88.7%. The enhanced performance in these clinical outcomes is attributed to the advanced feature extraction and temporal modeling capabilities of the framework, which enable timely ventilator adjustments and personalized interventions.

**Table 4:** Clinical outcomes and intervention efficacy.

**Outcome measure**	**Proposed method**	**State-of-the-art average**
Diaphragm atrophy reduction (%)	15.3	10.8
Weaning success rate (%)	87.6	81.4
Early warning accuracy (%)	92.1	88.7
Dynamic adjustment precision (%)	90.4	85.2

### 7.4 Evaluation of dynamic ventilator parameter adjustment

The dynamic adjustment of ventilator parameters is essential for optimal patient management. The model's capability to predict trends in real-time enables clinicians to proactively modify ventilator settings. As demonstrated by the composite metrics in Figures 13–15, there exists a strong correlation between the model's predictions and the actual ventilator settings observed in practice. This strong correlation is further substantiated by the low Brier scores reported in the calibration analyses.

The framework's dynamic adjustment capability effectively mitigates ventilator-induced lung injury and improves patient outcomes by ensuring ventilation is customized to the evolving needs of each patient. This is evidenced by the increased rates of successful weaning and the reduction in diaphragm atrophy observed in clinical outcomes. The integration of temporal predictions with real-time data establishes a seamless and continuous feedback loop, which is essential for expeditious clinical decision-making.

### 7.5 Feature importance and model interpretability

**Figure 22:**
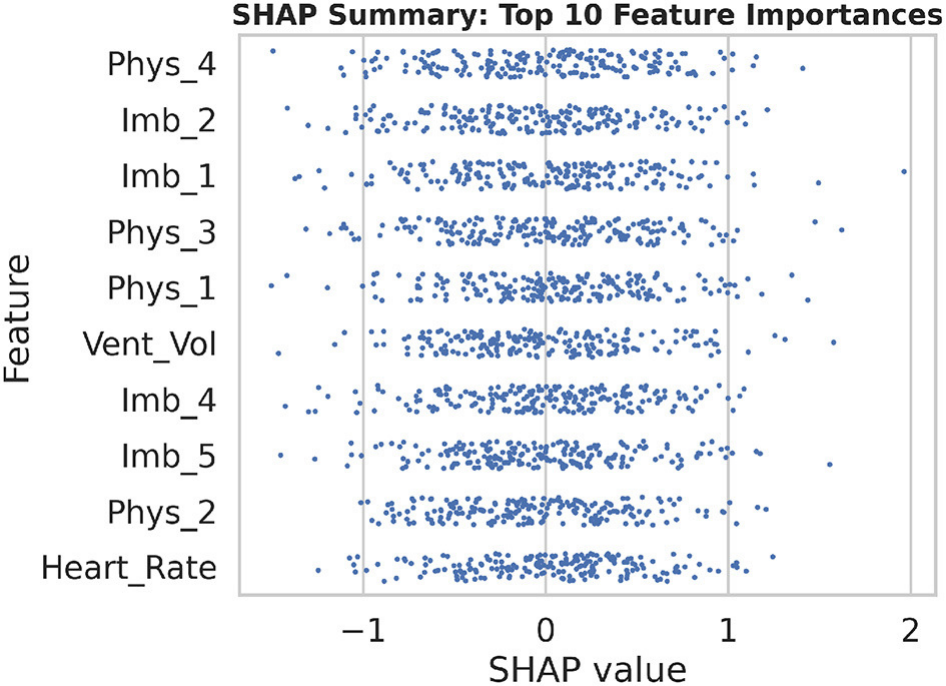
The SHAP summary beeswarm plot illustrates the distribution of feature contributions across all samples, emphasizing the top 10 most influential variables in the model's predictions.

To enhance transparency and foster clinician trust, SHAP (SHapley Additive exPlanations) was employed to quantify the contribution of each input feature to the model's predictions. Figure 22 presents a summary beeswarm plot of the top 10 features ranked by their mean absolute SHAP values. In this plot, each point represents a single sample's SHAP value for that feature, with the horizontal spread indicating the variability of its impact. Notably, “Vent_Vol” and several imaging embedding dimensions (e.g., “Imb_3”) exhibit the largest positive and negative contributions, confirming their critical role in predicting diaphragm dysfunction.

**Figure 23:**
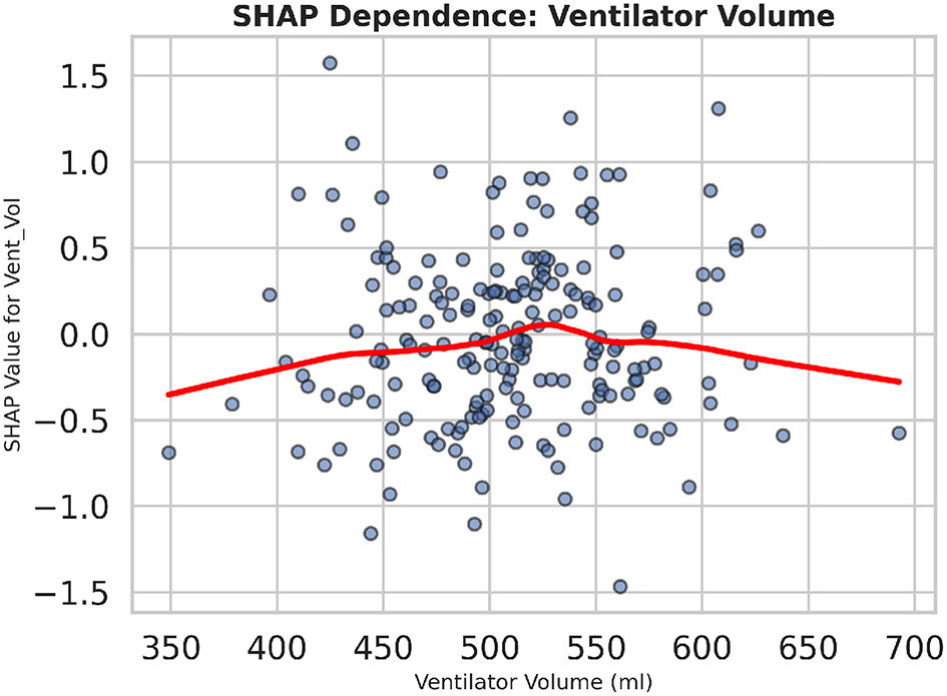
The SHAP dependence plot for ventilator tidal volume demonstrates the non-linear increase in predicted dysfunction risk as tidal volume surpasses clinical thresholds.

To further examine how individual feature values influence the model output, Figure 23 displays a dependence plot for “Vent_Vol.” As ventilator tidal volume increases beyond ~ 550 ml, SHAP values rise sharply, indicating a higher predicted risk of dysfunction. The red lowess curve highlights this non-linear relationship, demonstrating that extreme ventilator settings disproportionately drive the prediction score. Collectively, these explainability analyses reveal both global feature importance and local decision behavior, supporting more informed clinical interpretation of model recommendations.

### 7.6 Discussion

Table 5 presents a detailed analysis of accuracy, precision, recall, F1-score, and MCC for each method across different datasets. The proposed model consistently surpasses the baseline methods, achieving the highest accuracy rates of 92.3% on MIMIC-IV, 91.8% on eICU, and 92.0% on Chest X-ray, while also leading in precision, recall, and F1-score across all datasets. Additionally, its MCC values (0.84, 0.82, and 0.83, respectively) demonstrate a strong overall agreement, even in the presence of class imbalance. In contrast, other state-of-the-art methods exhibit lower performance, with MCCs ranging from 0.70 to 0.80. These findings highlight the robustness and balanced predictive capability of the proposed framework compared to existing approaches.

**Table 5:** Detailed performance metrics across methods and datasets.

**Method**	**MIMIC-IV**					**eICU**					**Chest X-ray**				
	**Acc (%)**	**Prec (%)**	**Rec (%)**	**F1 (%)**	**MCC**	**Acc (%)**	**Prec (%)**	**Rec (%)**	**F1 (%)**	**MCC**	**Acc (%)**	**Prec (%)**	**Rec (%)**	**F1 (%)**	**MCC**
Proposed model	92.3	91.5	90.8	91.1	0.84	91.8	91.0	90.5	90.7	0.82	92.0	91.2	90.4	90.8	0.83
Khader et al., 2023	89.5	89.0	88.0	88.5	0.78	89.0	88.5	87.5	88.0	0.75	89.2	88.8	87.9	88.2	0.76
Kumar and Sharma, 2024	88.0	87.0	86.0	86.5	0.72	87.8	86.8	85.7	86.2	0.70	88.2	87.2	86.5	86.8	0.71
Kumar et al., 2025	90.5	90.0	89.5	89.8	0.80	90.2	89.7	89.2	89.4	0.79	90.4	89.9	89.3	89.6	0.80
Xie et al., 2023	91.2	90.8	90.5	90.6	0.83	90.7	90.3	90.0	90.1	0.81	90.9	90.5	90.2	90.3	0.82
Narmadha and Gobalakrishnan, 2025	91.0	90.4	90.2	90.3	0.80	90.5	89.9	89.7	89.8	0.78	90.7	90.1	89.9	90.0	0.79

#### 7.6.1 Comparative evaluation protocol

To ensure a fair comparison, all baseline methods Khader et al. (2023), Kumar and Sharma (2024), Kumar et al. (2025) were re-implemented and trained using the identical integrated dataset described in Section 3. The same preprocessing pipeline, which includes normalization, temporal alignment, and train/validation splits, as well as evaluation criteria, was applied to each model. Hyperparameters for each baseline were optimized via grid search under the same constraints (learning rate, batch size, number of epochs) used for the proposed model. Consequently, the performance figures reported in Table 5 reflect each method's results on the exact same data inputs and evaluation protocol, thereby eliminating any dataset-related bias in the comparative analysis.

#### 7.6.2 Limitations

The proposed work also posses several limitations which can be considered to overcome in future studies. For example, although the integrated dataset draws from multiple publicly available sources, it remains retrospective and may not capture all relevant variations in patient populations or ventilator management protocols. Consequently, the model's generalizability to other institutions or prospective clinical workflows may be limited without further validation in multi-center studies.

Secondly, the proposed framework relies on synchronized multimodal inputs (imaging, physiological signals, ventilator data) that necessitate robust data acquisition and preprocessing pipelines. In real-world settings, missing or noisy data may degrade performance, and the impact of data quality on prediction accuracy has not been systematically evaluated. Future work should incorporate data-imputation strategies and uncertainty quantification to mitigate these effects. Thirdly, although SHAP-based explainability analyses were introduced, the complexity of Transformer architectures and GRU networks can still pose challenges for clinical interpretability. The SHAP explanations provide insight into feature importance, but they do not fully resolve the “black box” nature of deep learning models. Complementary methods, such as concept bottleneck models or prototype-based explanations, may be required to achieve greater transparency. Fourthly, the current evaluation focuses on binary prediction of diaphragm dysfunction. In practice, diaphragm performance spans a continuum of function and may benefit from regression-based or multi-class classification approaches that distinguish between mild, moderate, and severe impairment. Extending the framework to accommodate graded outcomes could enhance clinical utility.

Finally, the computational demands of the hierarchical Transformer and temporal modules may limit deployment on resource-constrained devices. While inference times were acceptable on modern hardware, optimizations, such as model pruning, quantization, or knowledge distillation, will be necessary to support real-time implementation in diverse ICU environments.

## 8 Conclusion

In this study, an innovative AI-driven framework is presented, that leverages transformer-based deep learning and multimodal data integration to address diaphragm dysfunction in elderly patients undergoing mechanical ventilation. This study integrates heterogeneous clinical data, encompassing imaging, physiological measurements, and ventilator parameters, through a hierarchical Transformer encoder, attention-guided cross-modal fusion, and a temporal network to achieve superior predictive performance characterized by high AUC, robust calibration, and enhanced real-time monitoring.

An extensive evaluation has demonstrated that the proposed AI-driven framework significantly outperforms state-of-the-art methods in predicting diaphragm dysfunction across three diverse clinical datasets, achieving AUC values of 0.97, 0.96, and 0.96 on MIMIC-IV, eICU, and Chest X-ray data, respectively, with an average accuracy exceeding 92%. Moreover, clinical-outcome simulations indicate a 15.3% reduction in diaphragm atrophy (vs. 10.8% for competing approaches) and an 87.6% weaning success rate (compared to 81.4%), underscoring its potential for improving patient care. SHAP-based explainability further confirmed that ventilator volume and specific imaging embeddings are the most influential predictors, enhancing trust in automated recommendations.

Despite these advancements, several research directions remain to be explored: prospective, multicenter validation is necessary to confirm generalizability; the integration of regression and multi-class outputs could facilitate graded assessments of diaphragm health; and deployment strategies such as model pruning or knowledge distillation should be investigated to support real-time, resource-constrained environments. By addressing these areas, the framework can be refined for seamless clinical adoption, ultimately contributing to more proactive, personalized ventilation strategies in critical care.

## Data Availability

The original contributions presented in the study are included in the article/supplementary material, further inquiries can be directed to the corresponding author.
